# Decitabine With or Without Micro-Transplantation for the Treatment of Intermediate or High-Risk Myelodysplastic Syndrome: A Chinese Single-Center Retrospective Study of 22 Patients

**DOI:** 10.3389/fonc.2021.628127

**Published:** 2021-03-31

**Authors:** MinMing Li, Chao Li, SuXia Geng, XiaoMei Chen, Ping Wu, ChengXin Deng, XiaoFang Chen, ZeSheng Lu, JianYu Weng, Xin Du

**Affiliations:** ^1^Department of Hematology, Guangdong Provincial People's Hospital, Guangdong Academy of Medical Sciences, Guangzhou, China; ^2^Department of Hematology, School of Medicine, Guangdong Provincial People's Hospital, Guangdong Academy of Medical Sciences, South China University of Technology, Guangzhou, China

**Keywords:** myelodysplastic syndromes, HLA-mismatched micro-transplantation, decitabine, overall survival, chronic myelomonocytic leukemia

## Abstract

The treatment outcomes of intermediate or high-risk myelodysplastic syndrome (MDS) remain unsatisfactory. This study was designed to evaluate the safety and efficacy of human leukocyte antigen (HLA)-mismatched hematopoietic stem cell micro-transplantation (MST) in patients with MDS. A total of 22 patients with MDS, ranging between the ages of 39 and 74, were enrolled in this study. Eleven patients were given decitabine (DAC), a DNA methyltransferase inhibitor, combined with HLA-mismatched MST (MST-DAC group), and the remaining patients were given decitabine only (DAC group). The median overall survival (OS) of the MST-DAC group was higher than that of the DAC group (24 vs. 14.3 months; HR 0.32; 95% CI: 0.11–0.96; *p* = 0.04), although it is a study with small samples. The overall response rate (ORR), marrow complete remission (mCR), plus hematological improvement (HI) rates of the MST-DAC group were higher than that of the DAC group (81.8 vs. 54.5%, *p* = 0.36; 63.6 vs. 27.3%, *p* = 0.09, respectively); however, there were no statistical differences between the two groups, which may be attributed to the limited number of cases evaluated in this study. No graft-vs.-host disease was observed in the MST-DAC group. Patients in the MST-DAC group demonstrated a slightly lower incidence of hematological and non-hematological adverse events (AEs). DAC combined with HLA-mismatched MST may provide a novel, effective, and safe treatment for use in intermediate or high-risk MDS pathologies.

## Introduction

Myelodysplastic syndrome (MDS) represents a group of heterogeneous myeloid clonal diseases that originate from hematopoietic stem cells and are characterized as having an abnormal development of myeloid cells. Typically, MDS manifests as ineffective hematopoiesis and refractory cytopenia with the risk of transforming into acute myeloid leukemia (AML) (1). It is known that allogeneic hematopoietic stem cell transplantation (allo-HSCT) promotes curative effects in patients with MDS; however, clinicians often face impediments to its widespread application, particularly concerning infectious and other toxicities associated with conditioning regimens, the development of significant graft-vs.-host disease (GVHD) with resultant organ dysfunction, infection from prolonged immunosuppression, and in some cases, a high rate of disease progression (2).

Hypermethylation in DNA is associated with tumor progression and differentiation arrest, which has been detected in myelodysplastic syndrome (MDS) and AML (3, 4). Decitabine (DAC), a DNA hypomethylating agent, is considered a frontline therapy for intermediate or high-risk patients who were not candidates for allo-HSCT according to National Comprehensive Cancer Network (NCCN) Guidelines (Version 2.2020) for myelodysplastic syndromes. However, the clinical efficacy of demethylation drugs to treat patients with MDS remains limited.

Currently, several studies have shown that human leukocyte antigen (HLA)-mismatched hematopoietic stem cell micro-transplantation (MST) can increase complete remission (CR) rates, improve OS, and avoid the development of graft-vs.-host disease (GVHD) in patients with AML (5–7). The term MST refers to standard chemotherapy combined with a granulocyte colony-stimulating factor (G-CSF) mobilized peripheral blood stem cell (G-PBSCs) infusion of HLA-mismatched donor cells without the use of immunosuppressive agents (7). Although MST mediates graft-vs.-leukemia (GVL) effects and GVHD hardly occurs, it is unclear whether treatment with MST in combination with DAC can improve the outcomes of patients with intermediate or high-risk MDS compared with those treated with DAC-only. Thus, a retrospective study was designed to evaluate the safety and efficacy of DAC combined with or without MST in patients with MDS.

## Materials and Methods

### Patients

Data were retrospectively collected from 22 patients with MDS [WHO 2008 classification (8)] who were treated with MST-DAC or DAC at the Department of Hematology of Guangdong Provincial People's Hospital from 2006 to 2016. All of them did not have severe infection, liver and kidney dysfunction, or an uncured secondary tumor.

### Donors

Donors were selected based on the degree of HLA mismatched loci. The sex, age, and red blood cell type were not heavily considered when selecting donors.

Donor and recipient HLA-A, -B, -C, -DRB1, and-DQB1 loci were typed at intermediate resolution using polymerase chain reaction (PCR) paired with the sequence-specific primer method. Of the 11 patient/12 donor pairs, the HLA alleles of five donors were matched in 5 of 10, of three donors were matched in 0 of 10, of two donors were matched in 2 of 10, of one donor were matched in 3 of 10, and of one donor were matched in 6 of 10. The median age of the donors was 28 and included 6 adult-offspring donors, 2 relatives, and 4 unrelated donors (Supplementary Table 1).

The protocol was approved by the Human Ethics Committees of the Guangdong General Hospital, Guangdong Academy of Medical Science. The study was performed following ethical standards set forth by the Declaration of Helsinki. All patients and donors provided written informed consent before enrollment in the study.

### Data Collection

The data collected for analysis included the clinical characteristics of the patients, such as age at diagnosis, sex, past medical history of malignancies and/or hematological diseases, family history of malignancies and/or hematological diseases, performance status (PS), complete blood counts (CBCs), blasts in peripheral blood (PB) and bone marrow (BM), cellularity of BM, chromosome abnormalities, French–American–British (FAB) and WHO classifications, risk groups in Revised International Prognostic Scoring System (IPSS-R), treatments, date of initial therapy, date of progression to AML, and date of death or that of the last follow-up.

### Study Endpoints

The co-primary endpoints in this study included the overall response rate (ORR), overall survival (OS), and progression-free survival (PFS). The ORR included the rate of CR, partial response (PR), marrow complete remission (mCR), and hematological improvement (HI). The response was assessed according to the International Working Group (IWG) criteria (9). The OS was defined as the time from initiation of treatment to the date of death from any causes or until the end of the follow-up period. The PFS was defined as the time from initiation of medication to treatment failure, progression of the disease, or death from any causes. All MDS cases were confirmed based on local investigator reports and/or death certificate information. The duration of the follow-up period was measured as the date of the first treatment dose received for each patient up to 2 years.

### Safety

All severe (grade 3 or higher) hematological or non-hematological adverse events (AEs) that occurred during treatment were evaluated according to the Common Terminology Criteria for Adverse Events (CTCAE) v4.03 (10).

### Assessment of Wilms' Tumor Gene (WT1)

Wilms' tumor gene transcripts were quantified by a standard European LeukemiaNet real-time quantitative PCR using the ABI 7500 real-time PCR system (11).

### Mobilization and Apheresis of Donor Peripheral Mononuclear Cells

Apheresis of HLA-mismatched donor peripheral mononuclear cells was performed with a COBE-spectra 6.0 blood cell separator after the donor was subcutaneously injected with 5 μg/kg G-CSF two times a day for 5 days. The median numbers (range) of mononuclear and CD3+ cells infused per course were, respectively, 2.50 (0.97 ~ 4.08) × 10^8^ and 0.86 (0.79 ~ 1.02) × 10^8^ cells per kg. (Supplementary Table 2).

### Statistical Analysis

Variables related to clinical characteristics between the two groups were compared using Fisher's exact test. All time-to-event analyses were performed with the use of Kaplan–Meier methods and presented by Kaplan–Meier curves. The hazard ratio (HR) and 95% confidence intervals (CIs) were estimated in comparison to a reference risk of 1.0. Statistical significance was defined with a 2-sided *p* < 0.05. SPSS (version 25.0) was used for all statistical analyses. GraphPad Prism 7.0 was used for graphing the results.

## Results

### Patients

Of all the analyzed patients, 11 (11/22, 50.0%) were treated with MST-DAC and the rest (11/22, 50.0%) were treated with decitabine-only. In the MST-DAC group, the median patient age was 60 years (age range: 39–73 years) and 8 (72.6%) were female. The median age of the patients in the DAC group was 61 years (age range: 41–74 years) and 8 (72.6%) were female.

### Treatment

The DAC group received DAC treatment, which involved infusions of 20 mg/m^2^ DAC *via* intravenous drip on days 1–5. The patients in the MST group were also given an infusion of HLA-mismatched G-PBSCs within 24–72 h until the end of DAC treatment (20 mg/m^2^). When ANC was <0.5 × 10^9^/L, G-CSF at 5 μg/kg/day was subcutaneously administered. When hemoglobin was <60 g/L, an infusion of red blood cells was given, and when platelet count was <20 × 10^9^/L, an infusion of platelets was administered. Notably, there were no statistical differences in the number of DAC treatment cycles between the two cohorts. The median number of treatment cycles for the DAC group was 4 (range: 2–20) and for the MST-DAC group was 5 (range: 0–11) (*p* = 1.00) (Table 1). The median number of treatment cycles for the MST-DAC group, those who underwent micro-transplantation, was 4 (range: 2–4).

**Table 1:** Patient demographics and clinical characteristics.

**Characteristic**	**MST-DAC group (n** **=** **11)**		**DAC group (*****n*** **=** **11)**		**Fisher exact test value**	**Exact Sig. (2-sided)** ***P-value***
	**No. of patients**	**%**	**No. of patients**	**%**		
Sex					–	1.00
Male	8	72.6	8	72.6		
Female	3	27.4	3	27.4		
Age, years					–	1.00
Median	60		61			
Range	39–73		41–74			
≥60 years	7	63.6	7	63.6		
<60 years	4	36.4	4	36.4		
FAB classification					4.32	0.41
CMML-1	0	0	2	18.2		
CMML-2	1	9.1	1	9.1		
RAEB-1	4	36.4	6	54.5		
RAEB-2	2	18.2	1	9.1		
RCMD	4	36.3	1	9.1		
IPSS-R					1.14	1.00
Mediate (3–4.5 points)	3	27.3	4	36.4		
High (4.5–6 points)	7	63.6	7	63.6		
Very high (6 points)	1	9.1	0	0.0		
Therapy Times	DAC times %		DAC times%		1.24	0.73
Median	5		4			
Range	0–11		2–20			
<4	5	45.5	5	45.5		
≥4	6	54.5	6	54.5		
Donor/recipient with HLA mismatched loci						
6/10	1	9.1	–	–		
5/10	5	45.4	–	–		
3/10	1	9.1	–	–		
2/10	1	9.1	–	–		
NA	3	27.3	–	–		
WHO PS					–	1.00
1	11	100	11	100		

### Treatment Response

Of the nine patients (9/11, 81.8%) in the MST-DAC group who responded to treatment: seven achieved both mCR and HI (7/11, 63.6%), one had only mCR (1/11, 9.1%), and one had only HI (1/11, 9.1%). Responses were observed for all six (6/11, 54.5%) patients in the DAC group: three achieved both mCR and HI (3/11, 27.3%), and three had only mCR (3/11, 27.3%). Though no significant difference was observed between the two groups, the ORR and the ratio of achieving both mCR and HI in the MST-DAC group was higher than that for the DAC group (81.8 vs. 54.5%, *p* = 0.36; 63.6 vs. 18.2%, *p* = 0.09). The incidence of AML transformation within 12 months for the MST-DAC group was lower than that for the DAC group (0.0 vs. 27.3%, *p* = 0.21). Also, the incidence of death within 24 months for the MST-DAC group was lower than that for the DAC group (45.5 vs. 81.8%, *p* = 0.18).

### Survival Analysis

**Figure 1:**
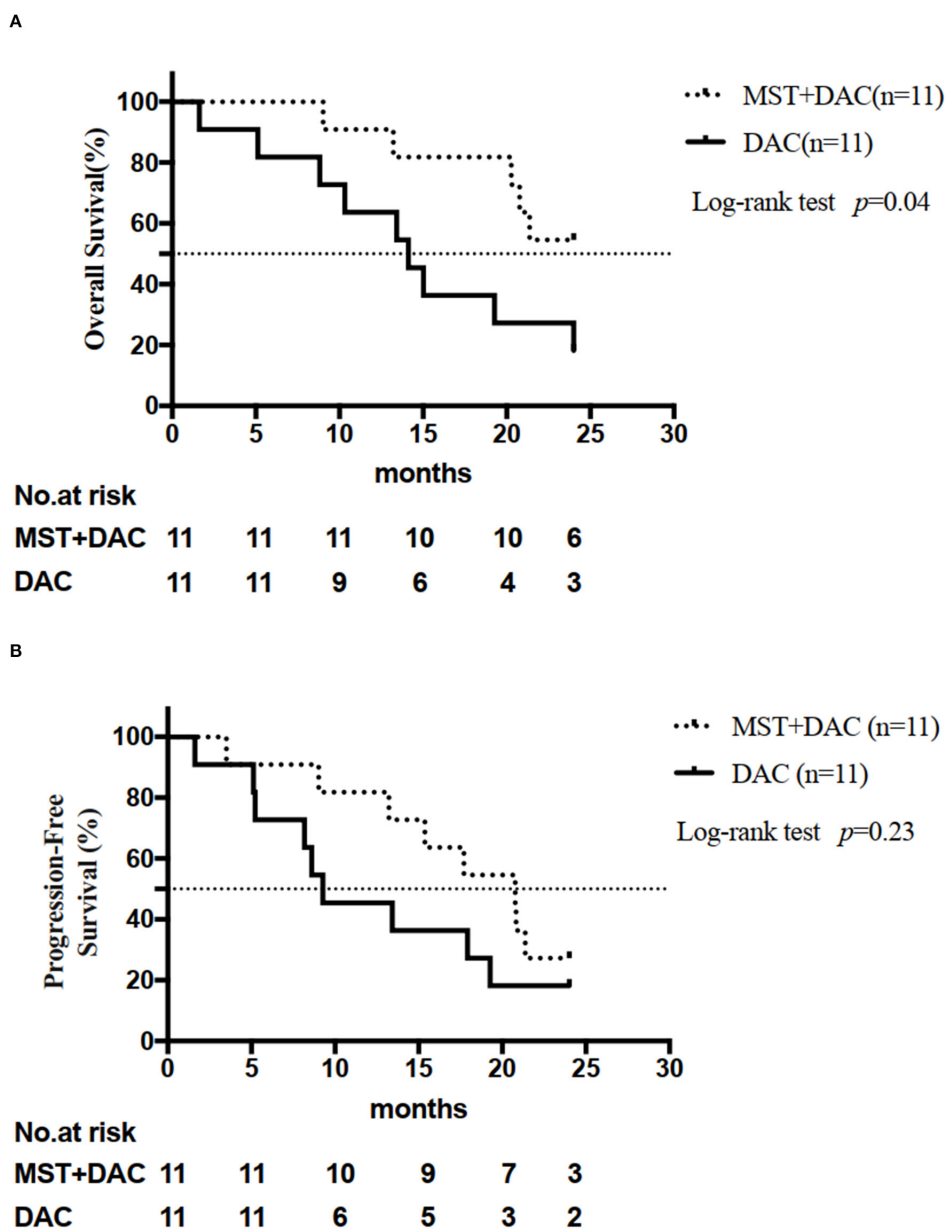
Analysis of efficacy endpoints. **(A)** Overall survival (OS) for the two groups is shown. **(B)** Progression-free survival (PFS) for the two groups is shown.

Six patients were still alive at the end of the follow-up period in the MST-DAC group and two patients were alive in the DAC group. The median OS was 24 months for the MST-DAC group and 14.13 months for the DAC group. A significant difference was observed between the two groups (HR: 0.32; 95% CI: 0.11–0.96; log-rank test, *p* = 0.04) (Figure 1A). The median PFS was 20.8 months for the MST-DAC group vs. 9.3 months for the DAC group (HR: 0.55; 95% CI: 0.20–1.47; log-rank test, *p* = 0.23) (Figure 1B and Supplementary Table 4).

### Safety and Toxicities

Safety profiles were evaluated for all patients in the cohort. During the first 12 months of treatment, one patient (1/11, 9.1%) died in the MST-DAC group, and four patients (4/11, 36.4%) died in the DAC group (*p* = 0.31). The mortality at 24 months was 5/11 (45.5%) for the MST-DAC group and 9/11 (81.1%) for the DAC group (*p* = 0.18) (Table 2).

**Table 2:** Patient outcomes.

**Response by IWG 2006 criteria**	**MST-DAC group**		**DAC group**		**Fisher exact *sig*.** **(2-sided)** ***P-value***
	***N***	**%**	***N***	**%**	
CR	0	0.0	0	0.0	-
mCR + HI	7	63.6	3	27.3	0.09
mCR only	1	9.1	3	27.3	
HI only	1	9.1	0	0.0	-
SD	2	18.2	3	27.3	-
Failure	0	0.0	1	9.1	-
Unable to evaluate	0	9.0	1	9.1	-
ORR (mCR, HI, CR)	9	81.8	6	54.5	0.36
Cumulative incidence of ORR					
At 2nd cycle	7	63.6	3	27.3	0.09
At 4th cycle	9	81.8	5	45.5	0.18
At 6th cycle	9	81.8	5	45.5	0.18
12-month incidence of AML transformation (%)	0	0.0	3	27.3	0.21
24-month incidence of AML transformation (%)	2	18.2	3	27.3	1.00
Cumulative incidence of AML transformation (%)	2	18.2	3	27.3	1.00
12-month incidence of death (%)	1	9.1	4	36.4	0.31
24-month incidence of death (%)	5	45.5	9	81.8	0.18
Cumulative incidence of death (%)	5	45.5	9	81.8	0.18

The most common AEs were neutropenia, thrombocytopenia, anemia, febrile neutropenia, leukopenia, septicemia, upper respiratory tract infection, hemorrhage, and pneumonia (Table 3). In general, patients in the MST-DAC group demonstrated a lower incidence of hematological AEs (anemia: 27.2% in the MST-DAC group vs. 55.0%, *p* = 0.39; leukopenia: 54.5 vs. 72.7%, *p* = 0.66; neutropenia: 55.0 vs. 64.0%, *p* = 1.00) during the entire treatment, with the exception of thrombocytopenia (64% in the MST-DAC group *vs*. 27.0% in the DAC group, *p* = 0.09). The same result was found for non-hematological AEs, where a lower incidence of febrile neutropenia, hemorrhage, and upper respiratory tract infection was also observed in the MST-DAC group (Table 3). By comparing complete blood cell counts after micro-transplantation, we found that absolute neutrophils were partially recovered. Therefore, micro-transplantation may shorten the recovery time of the hematopoietic function and reduce the incidence of infectious complications, such as pneumonia.

**Table 3:** Severe (grade 3 or higher) hematological or non-hematological adverse events (AEs) from the two therapies.

	**MST-DAC**				**DAC**				
	**Grade 3**	**Grade 4**	**Grade 5**	**Total**	**Grade 3**	**Grade 4**	**Grade 5**	**Total**	**Fisher exact sig.** **(2-sided)** ***P-value***
Hematological AEs									
Anemia	1	2	-	3 (27.2%)	3	3	-	6 (54.5%)	0.39
Leukopenia	6	0	-	6 (54.5%)	5	3	-	8 (72.7%)	0.66
Neutropenia	1	5	-	6 (54.5%)	0	7	-	7 (63.6%)	1.00
Thrombocytopenia	5	2	-	7 (63.6%)	0	3	-	3 (27.3%)	0.09
Non-hematological AEs									
Febrile neutropenia	3	1	0	4 (36.4%)	6	0	0	6 (54.5%)	0.40
Pneumonia	2	0	0	2 (18.2%)	5	0	1	6 (54.5%)	0.18
Septicemia	-	1	0	1 (9.1%)	-	0	1	1 (9.1%)	1.00
Upper respiratory tract infection	2	0	0	2 (18.2%)	4	0	0	4 (36.4%)	0.64
Hemorrhage	1	1	0	2 (18.2%)	0	2	2	4 (36.4%)	0.64
Soft tissue infection	1	0	0	1 (9.1%)	1	0	0	1 (9.1%)	1.00

### Wilms' Tumor Gene in Bone Marrow (BM-WT1)

Of the 22 patients in this study, 18 had detectable BM-WT1 before and after the treatment. The results demonstrated that among the 14 patients with a clinical response, the levels of BM-WT1 in 10 patients had different degrees of decline, and the median of the decline was 85.3% (range: 52.7–99.7%). The fluctuations of BM-WT in four patients were within the normal range, and there was no significant trend in the fluctuations for both cohorts with BM-WT1. Four patients failed to respond to therapy, while the levels of BM-WT1 in three of them who achieved SD remained unchanged. The levels of BM-WT1 in one patient remained to be higher than the normal range. Four patients with detectable BM-WT1 had a significant increase in BM-WT1 when progressed to AML (Supplementary Table 3).

## Discussion

Recently, Ai Huisheng et al. (6, 12–14) explored the application of “micro-transplantation” to treat several hematological malignancies, included MDS, AML, and Philadelphia chromosome-positive acute lymphoblastic leukemia. The rate of CR in patients with AML who received induction chemotherapy with mitoxantrone and cytarabine combined with HLA-mismatched G-PBSCs was 80%. The probabilities of the 2-year DFS and OS in an entire population were 38.9 and 39.3%, respectively (6). As reported by other published work that compared the efficacy of MST-DAC in treating MDS and transformed acute myelogenous leukemia (tAML), the ORR of patients with MDS treated with HLA mismatched MST-DAC combined with DAC and cytarabine was significantly higher than that of patients with tAML treated with HLA mismatched MST-DAC combined with DAC (81 vs. 50%; *p* = 0.03); the PFS and OS of 2 years were 42.7 and 84.7% in patients with MDS, respectively. No sign of acute and chronic GVHD was observed in patients during MST-DAC treatment (7). In another study of patients with MDS (*n* = 72) treated with MST (Microtransplantation-group, *n* = 28) or treated with two doses of DAC (DAC group, *n* = 27; low-dose DAC group, *n* = 17), the total CR rate was 42.9 vs. 14.8% and 29.4% in the three groups, respectively. The CR rate of the MST-group was significantly higher than that of the other groups (*p* = 0.02) (15). Results from clinical trials showed that the ORR of patients with MDS who received DAC fluctuates between 32 and 73% (16–19). In the present study, the ORR was 81.8% in the MST-DAC group, which was compared with 54.5% in the DAC group. Results from our study revealed that patients in the MST-DAC group showed a slightly higher ORR compared with the DAC group. Meanwhile, our retrospective analysis also suggested that a better OS was observed in patients who received MST-DAC (24.0 [9.0–24.0 months] vs. 14.3 months [1.6–24.0 months], *p* = 0.04). However, due to the small number of cases included in our retrospective study, future prospective trials with larger sample sizes are needed to verify our results.

According to the toxicities reported in our study, there was a lower incidence of severe hematological AEs in the MST-DAC group during the entire treatment period compared with the DAC only group: 27.2 vs. 54.5% for anemia (*p* = 0.39); 54.5 vs. 63.6% for neutropenia (*p* = 1,00); 54.5 vs. 72.7% for leukopenia, respectively (*p* = 0.66). A trend representing a lower percentage of febrile neutropenia, pneumonia, upper respiratory tract infection, and hemorrhage was also seen in the MST-DAC group. Compared with the DAC group, the incidences of AML transformation and the mortality rate were also lower in the MST-DAC group within 12 or 24 months (0.0 vs. 27.3%, 18.2 vs. 27.3%, 9.1 vs. 36.4%, 45.5 vs. 81.8%, respectively), which suggests that micro-transplantation was safe to treat patients with MDS. No signs of acute or chronic GVHD were observed in any of the patients during treatment, which reflects the same results reported in previous studies (6, 7, 13, 14). Our results illustrate the safety of the application of micro-transplantation combined with DAC treatment in patients with intermediate or high-risk MDS.

The purpose of micro-transplantation is to elicit an anti-tumor response, with little or no continuous donor cell implantation, no complete donor chimerism, and no onset of GVHD. Studies have shown that it is possible to obtain an anti-tumor response when only achieving microchimerism (<1% of donor cells) (20–25). It is speculated that T-cell and natural killer (NK)-cell alloreactivity could generate immediate anti-leukemic effects that awaken the anti-tumor immunity of the host, change the tolerance of the host to the tumor, and allow the host to undergo an immune response to the tumor (24, 26–30).

According to some reports, the mRNA level of WT1 reflects disease changes and progression in patients with MDS (31, 32). Therefore, WT1 is a suitable marker for the detection of minimal residual disease after SCT or chemotherapy (33). Furthermore, the correlation of WT1 mRNA levels before treatment and response was evaluated in the present study. There was a trend that indicated that the reduction of WT1 mRNA levels correlated with the efficacy of MST-DAC treatments (Supplementary Table 3).

The MST-DAC group included four relatively young (<60 years) patients who waited for suitable donors to undergo allo-HSCT, one patient achieved both mCR and HI, one patient achieved mCR only, and the other two patients achieved SD, and there was no evidence of GVHD. Therefore, the efficiency and safety of MST-DAC in relatively young patients who waited to undergo allo-HSCT was seen in this study. Patients who are candidates for allo-HSCT may be given MST-DAC as a bridging treatment for allo-HSCT.

According to the key eligibility criteria of micro-transplantation in our center, patients with blast <5% only received micro-transplantation without DAC, or with DAC for patients with blast ≥5%. In the MST-DAC group, two patients (MST-DAC 6 and MST-DAC 10) only received micro-transplantation without DAC and received supportive care pre-MST, and had planned to be given DAC if the disease was evolution. Both patients obtained marrow complete remission (MCR) after micro-transplantation, and one of them had received allo-HSCT after 4 years post-MST. We removed the data of the two patients, and the median OS of the MST-DAC group (*n* = 9) was still higher than that of the DAC group (*n* = 11) (24 vs. 14.1 months; HR 0.36; 95% CI: 0.12–1.07; *p* = 0.06). Although not statistically significant, there was a trend toward significance (*p* = 0.06) (Supplementary Figure 1).

In the current study, the overall survival of patients with MDS was effectively improved, in addition to the comparatively ORR, which makes our data noteworthy. At the same time, major drawbacks of our study include its retrospective design, the limited number of patients enrolled, and the long duration of the study (10 years). Due to the wide-range time of the study, the OS of patients could have been influenced by several factors which were listed in Supplementary Table 5. The median of the time from diagnosis to treatment for the MST-DAC group was 33 (range, 6–320) days, and that for the DAC group was 14 (range, 0–271)days. The median time of the duration of neutropenia/cytopenias before treatment for MST-DAC was 275 (range, 31–3605) days, and that for DAC was 230 (range, 20–2926).

In conclusion, our results are promising and show that MST combined with the probable synergistic effect of DAC can achieve a better OS in patients with intermediate or high-risk MDS and cause acceptable short-term toxicities. Prospective studies are urgently needed to determine the exact role of micro-transplantation in the setting of MDS and to clarify optimal treatment modalities, such as dosage and duration.

## Data Availability Statement

The original contributions presented in the study are included in the article/Supplementary Material, further inquiries can be directed to the corresponding author/s.

## Ethics Statement

The studies involving human participants were reviewed and approved by Ethics Committee of Guangdong General Hospital, Guangdong academy of Medical Science. The patients/participants provided their written informed consent to participate in this study.

## Author Contributions

All authors contributed to, revised, approved the manuscript content, and approved journal submission of the manuscript. The authors are fully responsible for all content and editorial decisions.

## Conflict of Interest

The authors declare that the research was conducted in the absence of any commercial or financial relationships that could be construed as a potential conflict of interest.
