# Supplementary material for: Decitabine With or Without Micro-Transplantation for the Treatment of Intermediate or High-Risk Myelodysplastic Syndrome: A Chinese Single-Center Retrospective Study of 22 Patients
Source: Front Oncol. 2021 Mar 31;11:628127. doi: 10.3389/fonc.2021.628127 (PMC8044401; doi:10.3389/fonc.2021.628127)
Supplement: Supplementary file 1 [file Data_Sheet_1.docx]

| Supplemental Table 1. Characteristics of Donors | | | | | | |
| --- | --- | --- | --- | --- | --- | --- |
| Patient NO. | Donor | Relationship | Donor age | Donor Sex | HLA locus | Blood type donor/receptor |
| MST-DAC 1 | donor 1 | related | 36 | male | 2/10 | B/AB |
| MST-DAC 2 | donor 1 | related | 25 | female | 5/10 | A/A |
| MST-DAC 3 | donor 1 | related | 40 | male | 5/10 | O/O |
| MST-DAC 4 | donor 1 | related | 23 | male | 5/10 | O/B |
| MST-DAC 5 | donor 1 | related | 27 | male | 0/10 | O/O |
| MST-DAC 6 | donor 1 | related | 38 | female | 5/10 | B/O |
| MST-DAC 7 | donor 1 | related | 28 | male | 5/10 | O/O |
| MST-DAC 8 | donor 1 | unrelated | 19 | male | 0/10 | A/A |
| MST-DAC 9 | donor 1 | unrelated | 33 | female | 0/10 | O/O |
| MST-DAC 10 | donor 1 | related | 28 | male | 6/10 | O/B |
| MST-DAC 11 | donor 1 | unrelated | 25 | male | 3/10 | A/B |
| MST-DAC 11 | donor 2 | unrelated | 34 | male | 2/10 | A/B |
| median | - | - | 28 | - | - | - |

| Supplemental Table 2. Characteristics of infused G-PBSC of Patients | | |
| --- | --- | --- |
| G-PBSC (cycle) | Median, /Kg | Range, /Kg |
| Mononuclear cells | 2.50 x10^8^ | (0.97 ~ 4.08 )x 10^8^ |
| CD34+cells | 2.03 x10^6^ | (1.06～2.99) x 10^6^ |
| CD3+ cells | 0.86 x10^8^ | (0.79～1.02) x 10^8^ |

| Supplemental Table 3. The levels of Wilms’ tumor gene (WT1) in bone marrow(BM) before and after therapy in Two cohorts. | | | | |
| --- | --- | --- | --- | --- |
| Patients No. | WT1 | | | Response by IWG 2006 criteria |
|  | Pre-treatment | Post-treatment | Progressed to AML |  |
| MST-DAC 1 | 81.6 | 32.16 | - | ﻿mCR + HI |
| MST-DAC 2 | 230.51 | 4.31 | - | ﻿mCR + HI |
| MST-DAC 3 | 42.77 | 8.79 | - | ﻿mCR + HI |
| MST-DAC 4 | 45.76 | 4.06 | - | ﻿mCR + HI |
| MST-DAC 5 | 0.18 | 0.23 | - | ﻿mCR + HI |
| MST-DAC 6 | - | - | - | ﻿mCR + HI |
| MST-DAC 7 | 44.46 | 12.26 | - | ﻿mCR + HI |
| MST-DAC 8 | - | - | - | ﻿SD |
| MST-DAC 9 | 400.58 | 347.47 | - | ﻿SD |
| MST-DAC 10 | 320.59 | 19.83 | - | ﻿mCR only |
| MST-DAC 11 | 1651.4 | 576.03 | 1834.06 | ﻿HI only |
| DAC 1 | 122.52 | 125.07 | - | ﻿SD |
| DAC 2 | - | - | - | ﻿SD |
| DAC 3 | 4.75 | 1.43 | - | ﻿mCR only |
| DAC 4 | 448.27 | 516.69 | - | ﻿SD |
| DAC 5 | 424.04 | 14.15 | - | ﻿mCR + HI |
| DAC 6 | 2761.67 | 1307.66 | 2921.12 | ﻿mCR only |
| DAC 7 | 7.53 | 1.75 | 130.76 | ﻿mCR + HI |
| DAC 8 | 13.69 | 3.08 | - | ﻿mCR only |
| DAC 9 | 3553.94 | 9.09 | 3536.54 | ﻿mCR + HI |
| DAC 10 | - | - | - | ﻿Unable to evaluate |
| DAC 11 | 1999.96 | 1329.69 | - | ﻿Failure |

| Supplemental Table 4. The Information of OS, PFS and PS in Two Cohorts | | | | | | | | | | | |
| --- | --- | --- | --- | --- | --- | --- | --- | --- | --- | --- | --- |
| Patients NO. | Sex | Age | WHO PS | ﻿Initiation of Medication | PFS Follow-up（24 months） | | |  | OS Follow-up（24 months） | | |
|  |  |  |  |  | Time of Disease Progression | PFS (Months) | Disease Progression 1:Yes 0:No |  | Time of Death | Survival Time (months) | Survival State 1:Dead from MDS 0:Alive, Lost or Dead from other reason |
| MST-DAC 1 | Male | 63 | 1 | 2014/12/30 | - | 27.43 | 0 |  | Alive | 24.00 | 0 |
| MST-DAC 2 | Male | 49 | 1 | 2015/7/8 | 2017/4/9 | 21.37 | 1 |  | 2017/4/9 | 21.37 | 1 |
| MST-DAC 3 | Male | 73 | 1 | 2015/7/1 | 2016/8/1 | 13.23 | 1 |  | 2016/8/1 | 13.23 | 1 |
| MST-DAC 4 | Male | 60 | 1 | 2014/12/22 | - | 24.00 | 0 |  | Alive | 24.00 | 0 |
| MST-DAC 5 | Male | 62 | 1 | 2016/1/18 | 2016/5/14 | 3.90 | 1 |  | Lost | 24.00 | 0 |
| MST-DAC 6 | Male | 65 | 1 | 2016/4/18 | 2018/1/1 | 20.77 | 1 |  | 2018/1/1 | 20.77 | 1 |
| MST-DAC 7 | Female | 61 | 1 | 2016/4/12 | 2017/12/28 | 20.83 | 1 |  | Alive | 24.00 | 0 |
| MST-DAC 8 | Female | 39 | 1 | 2016/8/10 | 2018/1/23 | 17.70 | 1 |  | Alive | 24.00 | 0 |
| MST-DAC 9 | Male | 39 | 1 | 2016/3/5 | 2016/12/1 | 9.03 | 1 |  | 2016/12/1 | 9.03 | 1 |
| MST-DAC 10 | Female | 54 | 1 | 2016/11/23 | - | 24.00 | 0 |  | Alive | 24.00 | 0 |
| MST-DAC 11 | Male | 60 | 1 | 2016/10/25 | 2018/1/29 | 15.37 | 1 |  | 2018/6/25 | 20.27 | 1 |
| DAC 1 | Female | 47 | 1 | 2013/6/24 | 2013/11/28 | 5.23 | 1 |  | 2014/9/18 | 15.03 | 1 |
| DAC 2 | Female | 56 | 1 | 2014/10/24 | 2015/12/1 | 13.43 | 1 |  | 2015/12/1 | 13.43 | 1 |
| DAC 3 | Female | 58 | 1 | 2010/6/13 | - | 24.00 | 0 |  | Alive | 24.00 | 0 |
| DAC 4 | Male | 61 | 1 | 2015/10/30 | 2016/8/3 | 9.27 | 1 |  | 2016/9/4 | 10.33 | 1 |
| DAC 5 | Male | 73 | 1 | 2010/9/9 | 2012/4/9 | 19.27 | 1 |  | 2012/4/9 | 19.27 | 1 |
| DAC 6 | Male | 41 | 1 | 2015/11/4 | 2016/7/6 | 8.17 | 1 |  | 2016/7/26 | 8.83 | 1 |
| DAC 7 | Male | 74 | 1 | 2009/11/19 | 2011/5/11 | 17.93 | 1 |  | 2011/11/14 | 24.00 | 1 |
| DAC 8 | Male | 63 | 1 | 2010/6/13 | - | 24.00 | 0 |  | Alive | 24.00 | 0 |
| DAC 9 | Male | 64 | 1 | 2014/6/26 | 2015/3/11 | 8.60 | 1 |  | 2015/8/24 | 14.13 | 1 |
| DAC 10 | Male | 71 | 1 | 2011/4/1 | 2011/5/20 | 1.63 | 1 |  | 2011/5/20 | 1.63 | 1 |
| DAC 11 | Male | 60 | 1 | 2014/10/1 | 2015/3/4 | 5.13 | 1 |  | 2015/3/23 | 5.77 | 1 |
| Note. PFS: Progression-Free Survival ; WHO PS: WHO ﻿Performance Status | | | | | | | | | | | |

| Supplemental Table 5. Other important characteristics of Patients. | | | | |  |  |
| --- | --- | --- | --- | --- | --- | --- |
| Group | Initiation of Dignosis | Initiation of Medication | Time from Diagnosis to Treatment（Days） | Duration of Neutropenia/cytopenias Before Treatment（Days） | Comorbidities | Causes of Death |
| MST-DAC 1 | 2014/12/24 | 2014/12/30 | 6 | 31 | Hypertension、Diabetes | Alive |
| MST-DAC 2 | 2015/6/5 | 2015/7/8 | 33 | 393 | α-thalassemia | Intracerebral haemorrhage |
| MST-DAC 3 | 2015/6/26 | 2015/7/1 | 5 | 275 | Diabetes | Infection |
| MST-DAC 4 | 2014/11/3 | 2014/12/22 | 49 | 229 | - | Alive |
| MST-DAC 5 | 2015/12/29 | 2016/1/18 | 20 | 200 | the surface antigen of hepatitis B virus is positive, Polyps of the colon, Atrophic gastritis | Lost |
| MST-DAC 6 | 2016/1/13 | 2016/4/18 | 96 | 1026 | - | Intracerebral haemorrhage |
| MST-DAC 7 | 2016/4/5 | 2016/4/12 | 7 | 67 | - | Alive |
| MST-DAC 8 | 2016/5/5 | 2016/8/10 | 97 | 817 | - | Alive |
| MST-DAC 9 | 2016/3/2 | 2016/3/5 | 3 | 303 | the surface antigen of hepatitis B virus is positive | Intracerebral haemorrhage |
| MST-DAC 10 | 2016/1/8 | 2016/11/23 | 320 | 3605 | α-thalassemia、atherogenesis | Alive |
| MST-DAC 11 | 2016/9/22 | 2016/10/25 | 33 | 183 | appendectomy、mild fatty liver 、Syphilis、Diabetes | Infection |
| DAC 1 | 2013/6/24 | 2013/6/24 | 0 | 20 | - | Intracerebral haemorrhage |
| DAC 2 | 2014/10/15 | 2014/10/24 | 9 | 39 | Cholelithiasis. | Infection |
| DAC 3 | 2010/6/7 | 2010/6/13 | 6 | 2926 | Uterine leiomyoma | Alive |
| DAC 4 | 2015/9/9 | 2015/10/30 | 51 | 636 | - | Infection |
| DAC 5 | 2010/8/24 | 2010/9/9 | 16 | 556 | Diabetes | Infection |
| DAC 6 | 2015/10/29 | 2015/11/4 | 6 | 1596 | - | Infection |
| DAC 7 | 2009/8/31 | 2009/11/19 | 80 | 230 | - | Infection |
| DAC 8 | 2010/6/9 | 2010/6/13 | 4 | 34 | Coronary artery disease | Alive |
| DAC 9 | 2014/6/12 | 2014/6/26 | 14 | 74 | Ulcerative colitis | Infection |
| DAC 10 | 2011/3/8 | 2011/4/1 | 24 | 204 | - | Intracerebral haemorrhage |
| DAC 11 | 2014/1/3 | 2014/10/1 | 271 | 1351 | - | Infection |

B.


Supplemental Figure 1. Analysis of efficacy endpoints. A. Overall survival (OS) for the two groups is shown. B. Progression-free survival (PFS) for the two groups is shown.
